# Positron Annihilation Spectroscopy Complex for Structural Defect Analysis in Metal–Hydrogen Systems

**DOI:** 10.3390/ma15051823

**Published:** 2022-02-28

**Authors:** Iurii Bordulev, Roman Laptev, Viktor Kudiiarov, Roman Elman, Alexander Popov, Denis Kabanov, Ivan Ushakov, Andrey Lider

**Affiliations:** 1 Division for Experimental Physics, National Research Tomsk Polytechnic University, 634050 Tomsk, Russia; laptevrs@tpu.ru (R.L.); kudiyarov@tpu.ru (V.K.); rre1@tpu.ru (R.E.); avipopov@mail.ru (A.P.); lider@tpu.ru (A.L.); 2 Research Nuclear Reactor Center, National Research Tomsk Polytechnic University, 634050 Tomsk, Russia; kabanoff@tpu.ru (D.K.); jiaozu@tpu.ru (I.U.)

**Keywords:** positron annihilation, defects, in situ, neutron activation, ^64^Cu, nuclear reactor, hydrogen-induced defects, thermal vacancies, metal–hydrogen systems, magnesium hydride

## Abstract

The current work is devoted to developing a system for the complex research of metal–hydrogen systems, including in an in situ mode. The system consists of a controlled gas reactor with a unique reaction chamber, a radioisotope positron source, and a positron annihilation spectroscopy complex. The use of the system enables in situ investigation of the defect structure of solids in hydrogen sorption–desorption processes at temperatures up to 900 °C and pressures up to 50 bar. Experimental investigations of magnesium and magnesium hydride during thermal annealing were carried out to approve the possibilities of the developed complex. It was shown that one cycle of magnesium hydrogenation–dehydrogenation resulted in the accumulation of irreversible hydrogen-induced defects. The defect structure investigation of the magnesium–hydrogen system by positron annihilation techniques was supplemented with a comprehensive study by scanning electron microscopy, X-ray diffraction analysis, and hydrogen sorption–desorption studies.

## 1. Introduction

The study and control of metal–hydrogen systems have several specific features associated with the high hydrogen diffusion mobility in metals and alloys, and its high reactivity. Hydrogen can interact with different complexes, including vacancy-type defects, impurity atoms, dislocations, intrinsic interstitial atoms, and grain boundaries [1,2,3,4,5,6,7,8,9]. In addition, hydrogen actively interacts with existing structural defects and induces the formation of many new defects [10,11,12,13,14,15]. The mechanisms of hydrogen’s effect on defects and the structural-phase state and mechanical properties of metallic materials have not been fully established despite numerous studies in this area. The development of new and improvement of known methods for controlling defects in metal–hydrogen systems is extremely urgent since there are still unresolved problems of the degradation of metals with hydrogen, and there is also the need to create new functional materials for operation in hydrogen-containing environments [1,2,3,4].

For the early detection of hydrogen embrittlement of metals and alloys, it is crucial to control the interaction of dislocations and hydrogen-vacancy complexes. In particular, it is necessary to study the mechanisms and dynamics of the appearance of defects, the transformation of one type into another, the reasons for their enlargement and disappearance, migration over surfaces, and the volume of the material under study. In addition, it is essential to establish the actual size and concentration of defects and identify the parameters influencing physical and mechanical properties. Positron spectroscopy methods are the most effective for monitoring the interaction of hydrogen with defects and revealing the formation mechanisms of hydrogen-induced defects. Furthermore, these methods are sensitive and can determine the type and concentration of defects and their chemical environment [1,2,3,4].

The effectiveness of positron spectroscopy for studying and monitoring metal–hydrogen systems has been demonstrated in many works [1,2,3,4,5,6,7,8,9,10,11,12,13,14,15,16,17,18,19,20]. However, there remain significant limitations associated with the impossibility of directly studying the evolution of the defect structure in the processes of absorption, accumulation, and distribution of hydrogen, despite the recent appearance of positron spectrometers and spectrometric complexes with high technical characteristics. Nevertheless, the study of defect formation during in situ investigation will make it possible to establish the primary mechanisms, stages, and principles of change in the structural-phase state of metal systems, which will make it possible to predict changes in physical and mechanical properties.

Consequently, the study of defects after exposure (ex situ) and directly in the process of exposure (heat treatment, hydrogenation, etc.) in the in situ mode becomes an urgent scientific task. However, this cannot be achieved using traditional methods of positron spectroscopy since it is necessary to locate the positron source directly in the research chamber under extreme conditions. Moreover, in such cases the positron source will also be negatively affected, which will lead to its destruction and radiation contamination of the entire system.

Thus, the purpose of this work is to develop a universal positron annihilation spectrometry complex for analysing thermal and hydrogen-induced defects in ex situ and in situ modes.

## 2. Materials and Methods

### 2.1. Positron Annihilation Spectroscopy Complex

**Figure 1:**
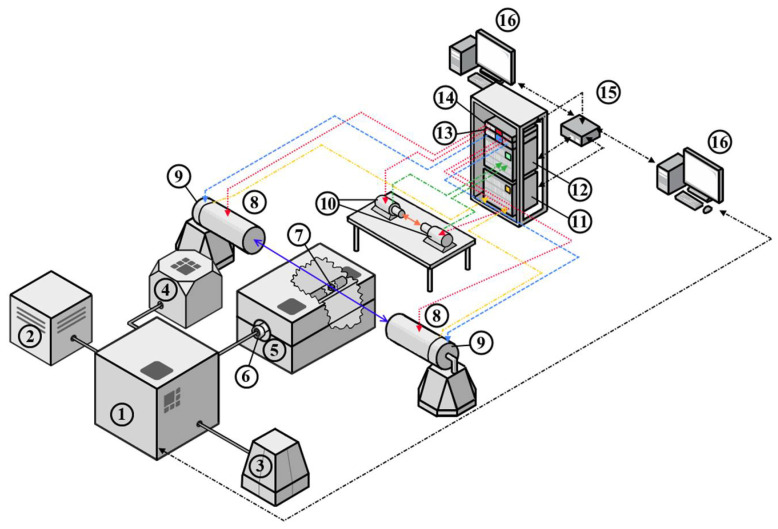
Diagram of a PAS complex to study changes in the defect structure of new functional materials when interacting with hydrogen: 1—controlled gas reactor, 2—hydrogen generator, 3—compressor, 4—vacuum pump, 5—high-temperature furnace, 6—chamber, 7—sample in the form of a “sandwich” geometry (sample-positron source-sample), 8—HPGe detectors, 9—HPGe preamplifiers, 10—scintillator detectors, 11—Doppler broadening spectroscopy (DBS) module APV8002, 12—positron annihilation lifetime spectroscopy (PALS) module APV8702, 13—preamplifier feeding APV4004, 14—high voltage unit APV3304, 15—network switchboard, 16—personal computers.

Considering the features and requirements for the analysis of metal–hydrogen systems, a digital positron annihilation spectroscopy (PAS) complex was developed, whose structural diagram is shown in Figure 1.

The complex combines a specially developed Sieverts-type automated apparatus and a spectrometric module. The Sieverts-type apparatus implements the interaction of solids with gases under different conditions (temperature, pressure, time). At the same time, there is a possibility to control conditions automatically and set the algorithm of conditions to change depending on the experiment’s stage. In addition to the automated system, the gas reactor includes a vacuum chamber for high pressure (up to 50 bar), a high-temperature furnace for controlled heating up to 900 °C, a sliding vane rotary pump for vacuum generation in the experimental chamber, a high-purity hydrogen source for hydrogen–solids interaction studies, a compressor to control the vacuum system, and a computer to control the gas reactor.

The standard procedure of hydrogenation using this complex is as follows. First, the sample is placed in a vacuum chamber installed in the furnace and connected to the controller. After vacuum is established, the chamber with the sample is heated to the specified temperature. Hydrogen from a generator is then pumped into the chamber to the required pressure. Finally, the pressure drop associated with hydrogen absorption by the sample in the chamber is recorded using specially developed software.

The hydrogen release energy is determined by thermodesorption spectroscopy experiments using an RGA100 mass spectrometer built into the controlled gas reactor. This makes it possible to obtain thermodesorption spectra after hydrogen saturation of the samples without extracting the sample from the vacuum medium. In this experiment, linear heating is carried out up to the temperature at which the hydrogen is released from the material. During the linear heating, hydrogen and other gases present in the sample are desorbed and monitored by a mass spectrometer. After the experiment is completed, the sample is cooled and removed from the chamber or re-saturated with hydrogen.

The spectrometric section from TechnoAP Co., Ltd. (Mawatari, Hitachinaka-shi, Ibaraki, Japan) consists of two functional modules: positron annihilation lifetime spectroscopy (PALS) and Doppler broadening spectroscopy (DBS), performed in a fully digital version. PALS uses the classical delayed coincidence method for the analysis [1,2,3]. The method consists of measuring the time between delayed coincidences of a nuclear γ-quantum associated with positron birth (the “Start” signal) and an annihilation γ-quantum with an energy of 0.511 MeV (the “Stop” signal). Scintillation (BaF) detectors carry out registration of γ-quanta. The detectors are powered by a four-channel high voltage power supply from TechnoAP, model APV3304. The APV3304 high voltage power supply is a VME module specifically designed to work with various nuclear radiation detectors. The output voltage is adjustable from 0 V to ±5000 V, and the maximum output current is 4 mA; hence, it is suitable for powering both PALS and DBS detectors. The high voltage power supplies are installed in the TechnoAP VME cradle model APV9007. The signals from the detector are fed directly to the high-speed, multi-channel data conversion system (TechnoAP APV8702 VME 8-bit, 2-channel digitizer, sampling rate up to 3 GS/s and bandwidth of 3 GHz). The spectrometry module and the high voltage power supply module are controlled, collected, and processed via an Ethernet link and a network switch to a PC using dedicated TechnoAP software from TechnoAP Co., Ltd.

The DBS method measures the energy distribution of annihilation electrons in the matter by measuring the energy shift from the nominal value 0.511 MeV. Application of the coincidence scheme allows significant reduction of the background, by about three orders of magnitude [1,2,3], and allows observation of the high-pulse part of the spectrum from the annihilation of positrons with core electrons. Furthermore, analysis of the high-pulse part of the spectrum makes it possible to determine the chemical composition at the site of positron annihilation. Therefore, coincidence Doppler broadening spectrometry (CDBS) is widely used to identify defects in various alloys and characterise small numbers of inclusions [1,2,3,4].

The γ-quanta are detected by two high-purity germanium (HPGe) semiconductor detectors. The detection unit combines a Model GC3018 solid-state detector, a Model 7600 SL low-background immersion cryostat, and a Model iPA-SL intelligent preamplifier for HPGe detectors from Canberra Industries, Inc. (now part of Mirion Technologies, Inc., Meriden, USA). The efficiency of such a detection unit is 30%, and the resolution is 0.875 keV and 1.80 keV for 122 and 1332 keV peaks, respectively.

The signals from the HPGe are routed to a high-speed, multi-channel data conversion system, which is a 14-bit, 2-channel VME digitizer, Model APV8002 from TechnoAP, sampling rate up to 100 Mbps, 100 MHz bandwidth. The digitizer also has a built-in coincidence circuit, allowing the possibility of realising the coincidence DBS mode when using two detectors.

A radioactive isotope is used as a source of positrons, positioned between two material samples and forming a so-called “sandwich geometry” [1,2,3,4]. Calibration and adjustment of ex situ measurements take place on a radioisotope source based on ^44^Ti β+ isotope, which is well-suited to both DBS and PALS studies because a nuclear γ-quantum with an energy of 1.157 MeV is emitted almost simultaneously with the positron. A positron source with an initial activity (30/09/2020) of 1.38 MBq (37.2 µCi) and maximum positron energy of 1.47 MeV was manufactured at Cyclotron CJSC.

A S-3400N scanning electron microscope (Hitachi, Tokyo, Japan) was used to analyse the microstructure of the obtained materials. Structural-phase analysis was performed on an XRD-7000S diffractometer equipped with a OneSight high-speed wide-angle detector (Shimadzu, Japan). Analysis of diffraction patterns and identification of phases were carried out using the PDF-4+ 2020 database (International Center for Diffraction Data, Newtown Township, PA, USA) and the PowderCell 2.4 (Federal Institute for Materials Research and Testing, Berlin, Germany) program. Hydrogen concentration measurements were performed on the hydrogen analyzer RHEN602 from LECO, St. Joseph, MI, USA. The studies using all the equipment mentioned above were conducted on the premises of Tomsk Polytechnic University.

### 2.2. Positron Source for In Situ Study

A copper isotope-based positron source was developed for in situ analysis of studied material [21].

This source was produced by irradiating pure (99.99%) and thin (10 μm) copper foil in the thermal neutron flux (4–5 × 10^13^ ns/cm^2^) of the IRT-T nuclear research reactor at Tomsk Polytechnic University.

The irradiation and subsequent soaking outside the neutron field were performed in such a way that the fast-living isotope ^66^Cu completely decayed, and the activity of the ^64^Cu isotope reached the value of about 60 MBq. Usually, for this purpose, a 5 mg copper foil was irradiated for 20–30 min, followed by exposure outside the neutron field for 20 h.

The resulting positron source was placed in a vacuum chamber (Figure 1) together with the investigated material. The in situ study allows DBS measurements to be carried out under aggressive conditions (high temperature, hydrogen atmosphere). In this study, in situ measurements were used to investigate the defect structure of Mg and MgH_2_ powder under high-temperature annealing conditions.

Both materials were first measured at room temperature and then annealed in a stepwise manner. The specified temperature profile included temperatures of 25, 250, 350, 400, and 450 °C. Annealing at each temperature was carried out for three hours. The heating rate between steps was equal to 5 °C/min. Each measurement (with and without heating) was carried out in situ in a vacuum (the residual pressure in the chamber at room temperature was around 6 mTorr). For each measurement, a new positron source was prepared.

Collected DBS spectra were processed with 15 min collection time. The line shape parameters (S and W) of the spectra were used to estimate the defect structure.

### 2.3. Materials for Testing

Milled MPF-4 magnesium powder of high purity (99.2%) with a particle size of 50–300 µm was used as the investigated material [22,23,24,25]. Magnesium hydride powder and dehydrogenated MgH_2_ powder were also used for testing. Magnesium hydride was obtained by hydrogenation from the gas phase using a specially developed Sieverts-type automated apparatus. Before hydrogenation, magnesium powder was mechanically activated in an AGO-2 planetary ball mill for 1 h in an argon atmosphere. The process of gas-phase hydrogenation was carried out at a temperature of 400 °C and pressure of 30 atm H_2_. Upon reaching the temperature and pressure values in the chamber, the magnesium powder was kept in the chamber for 5 h.

## 3. Results and Discussion

**Figure 2:**
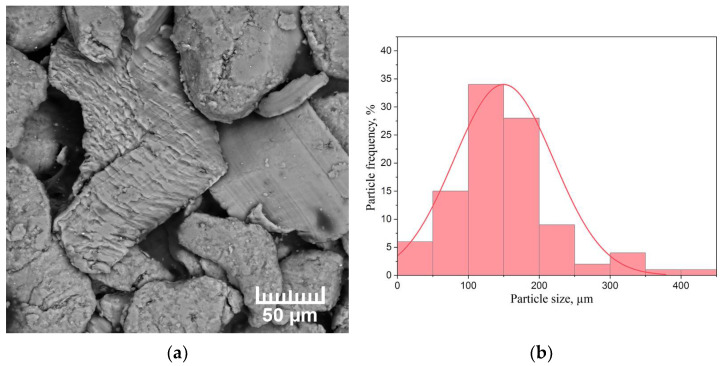
(**a**) SEM micrograph of the milled MPF-4 magnesium powder and (**b**) particle size distribution histogram.

As-received MPF-4 magnesium powder is unable to adsorb enough hydrogen to form a significant volume fraction of magnesium hydride due to the presence of a native oxide on the Mg surface. Therefore, in order to consider magnesium as a hydrogen storage material, the magnesium powder was mechanically activated in the AGO-2 planetary mill for 1 h. A scanning electron microscopy (SEM) micrograph of the milled magnesium powder selected for testing, as well as a histogram of the particle size distribution, are shown in Figure 2.

The powder consists of chip-like particles of irregular geometric shapes. The average particle size was 150 µm. The basic number of particles is characterised by the size of (90 ÷ 210) µm. The largest particles are 440 µm in size and there are also a certain number of particles less than 50 µm in size. The surface structure was investigated by the scanning electron microscope Hitachi S-3400N.

The ex situ PALS experimental results for magnesium powder at different annealing temperatures are shown in Table 1. The PALS spectra with the statistics of 5 × 10^6^ were analysed by the “four-state trapping model” using the LT10 software (version 10.2.2.2) from Institute of Material Science (University of Silesia), Katowice, Poland. In this model, the spectrum is analysed using four time components, τ_A_, τ_B_, τ_C_, and τ_F_; intensities, I_A_, I_B_, and I_C_; trapping rates, k_A_, k_B_, and k_C_; the average positron lifetime τ_avg_. The first three components correspond to the positron annihilation in A, B, and C states, while τ_F_ corresponds to the lifetime of a delocalized positron in the Mg lattice.

Table 1 shows that all lifetime spectra are well decomposed into four exponential components related to the annihilation of positrons at a different state in magnesium powder. The component with the shortest lifetime τ_F_ = 226 ± 1 ps corresponds to the positrons’ annihilation in a defect-free lattice of magnesium [26,27,28,29]. The second component with a lifetime of τ_A_ = 232 ± 1 ps is significantly lower than the values typical for monovacancies (253 ps [27,30], 292 ps [26]), and dislocations (244 ± 4 ps [28,31,32]). This component seems to be related to deformation defects (dislocations associated with twin boundaries [32]), which are typical for milled powders.

In addition, two long-lived components are distinguished in all spectra: τ_B_ = 392 ± 2 ps and τ_C_ = 2.4 ± 0.02 ns. The first appears to be associated with the annihilation of positrons trapped by the surface of the powder material. The positrons are thermalised in the powder and diffuse to the surface of a single powder particle. In this case, the lifetime is usually significantly increased compared to the annihilation of positrons in the defect-free lattice of the material under investigation [33]. The component with a lifetime of τ_C_ = 2.4 ± 0.02 ns is associated with the annihilation of orthopositronium localised in nanopores or in the space between the particles. Annealing to 300 °C does not lead to noticeable changes in PALS parameters. A further increase in temperature above 300 °C leads predominantly to annealing of dislocation defects.

**Figure 3:**
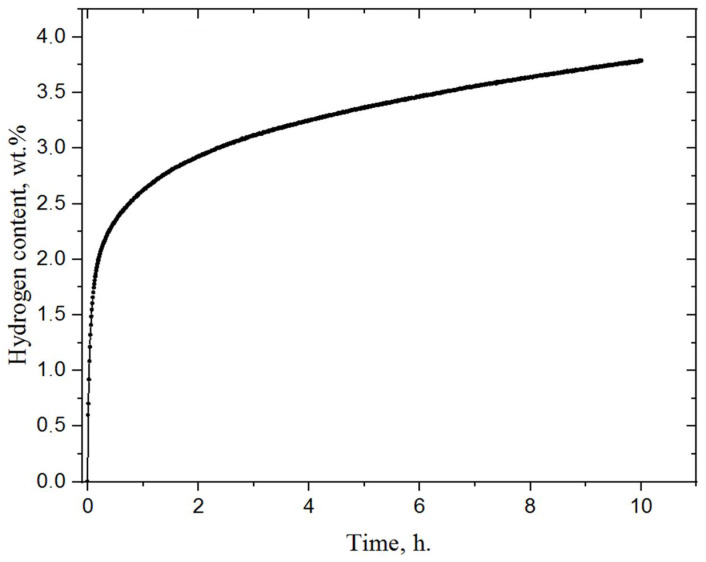
Hydrogenation curve of milled magnesium powder.

For further research, the milled magnesium powder was hydrogenated using a specially developed Sieverts-type automated apparatus at a temperature of 400 °C and pressure of 30 atm H_2_. The magnesium powder was left in the chamber in a hydrogen atmosphere for 5 h, after which the chamber slowly cooled to room temperature. At a pressure of 30 atm, mechanically-activated magnesium actively absorbs hydrogen, as a result of which the pressure in the chamber dropped to 23 atm. In this case, the sorption rate noticeably decreased, and the resulting sample contained only 64 vol.% of the hydride phase. In order to increase the volumetric content of magnesium hydride, hydrogen was again injected into the chamber with the powder to raise the pressure to 30 atm. This method of hydrogenation was tested in our other article [34]. After hydrogenation, the powder was milled in a planetary ball mill. This improves the desorption properties of material [35,36,37]. Figure 3 shows the hydrogenation curve of magnesium powder after activation. The temperature of hydrogenation was 400 °C and the initial pressure 30 atm.

The process of hydrogen sorption by magnesium powder at a temperature of 400 °C and pressure of 30 atm stays very active because of the high specific surface area [38,39,40]. It can be seen that activated magnesium absorbed 2.3 wt.% in half an hour, and then hydrogen absorption by magnesium slowed down. According to the data obtained, the activated magnesium powder absorbed about 3.7 wt.% within 10 h. Over the same time, the pressure in the chamber dropped from 30 to 23.6 atmospheres. The linear regions (in the first few minutes when hydrogen concentration increases dramatically) of these curves were used to obtain the sorption rate. According to calculations, the sorption rate for the first 10 min was about 7.5⋅10^−4^ cm^3^ (H_2_)/cm^2^⋅s.

**Figure 4:**
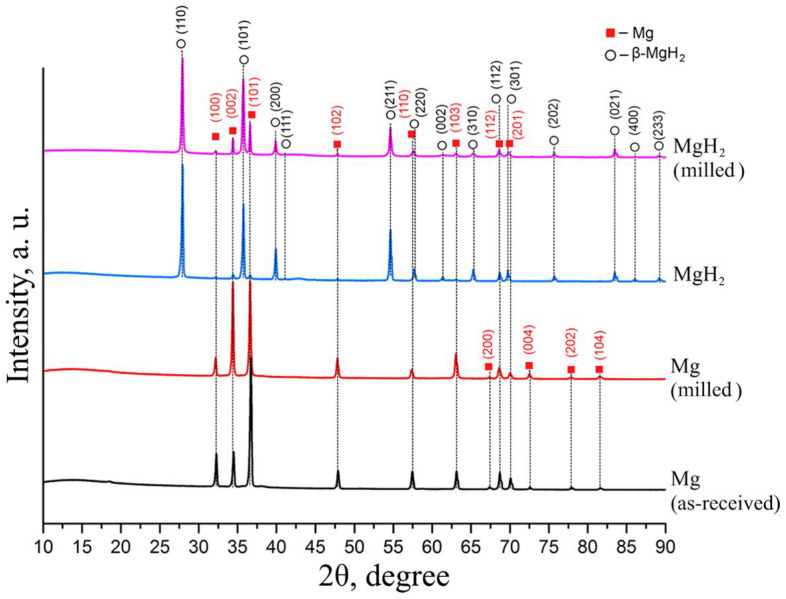
Diffraction patterns of magnesium and magnesium hydride.

The best result for magnesium conversion into hydride was achieved in a controlled gas reactor after four additional hydrogen injections of hydrogenation at a temperature of 400 °C and pressure of 30 atm H_2_. According to the results of X-ray phase analysis, the obtained powder consists of 99.2 vol.% MgH_2_ phase. Figure 4 shows the diffraction patterns of magnesium and magnesium hydride.

Analysis of the diffraction pattern presented above does not show the presence of other elements. Milling magnesium powder in a planetary mill leads to an increase in diffraction peaks corresponding to pure magnesium of the hexagonal close-packed (HCP) modification due to the destruction of particles and the formation of a “clean” magnesium surface. Hydrogenation leads to the formation of magnesium hydride β-MgH_2_ with a tetragonal rutile crystal structure [41,42]. Moreover, low-intensity reflections are retained, which corresponds to HCP α-Mg. The formation of a metastable orthorhombic γ-MgH_2_ phase was not observed. Milling magnesium hydride powder in a planetary ball mill does not lead to significant changes in the phase composition compared to magnesium hydride.

**Figure 5:**
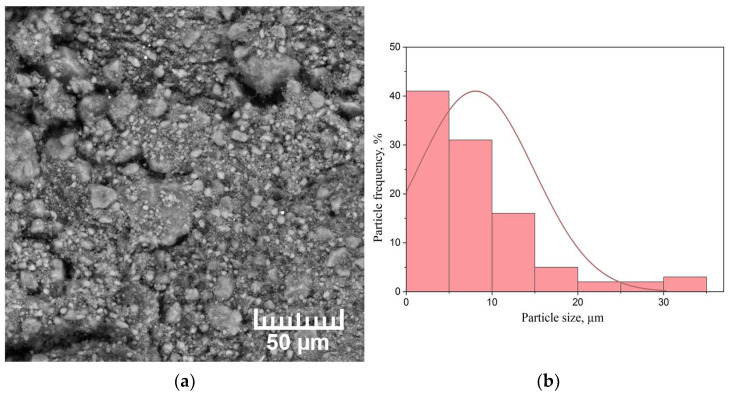
(**a**) SEM micrograph of the milled MgH_2_ powder and (**b**) particle size distribution histogram.

An SEM image of the milled magnesium hydride powder is shown in Figure 5.

The milling of MgH_2_ leads to a decrease in the average particle size to 9 µm; most of the particles are less than 5 µm. In addition, there are particles of about 30 μm in size, which are agglomerates of smaller particles.

According to data obtained using a gas analyzer, the hydrogen content in the milled magnesium hydride was 7.1 ± 0.4 wt.%. Considering that the theoretical hydrogen content in magnesium hydride is 7.67 wt.%, it can be concluded that as a result of saturation, most of the magnesium was transformed into a β-MgH_2_ phase.

Based on PALS data, an almost saturated positron trapping is characteristic for hydrogenated magnesium powder. The lifetime components are defined as follows: τ_A_ = 230 ± 1 ps (95.6%), τ_B_ = 515 ± 20 ps (2.8%), τ_C_ = 2.1 ± 0.1 ns (0.7%), τ_F_ = 219 ± 1 ps. The short-lived component τ_A_ = 230 ± 1 ps is slightly higher than the calculated values specific for magnesium hydride τ_MgH_2__ = 220 ± 1 ps [26,43], but much lower than the values typical of vacancy-type defects in this material τ_V(MgH_2_)_ = 245 ± 6 ps [26], which is apparently caused by a mixed state. The long-lived components τ_B_ = 515 ± 20 ps and τ_C_ = 2.1 ± 0.1 ns are also associated with positron annihilation on the powder surface and in nanopores, respectively. In the hydrogenated powder, the intensity of these components is significantly reduced compared to the milled magnesium powder (RT in Table 1). Noticeable changes in the magnesium powder before and after hydrogenation are observed on the ratio curves of the CDB spectra.

**Figure 6:**
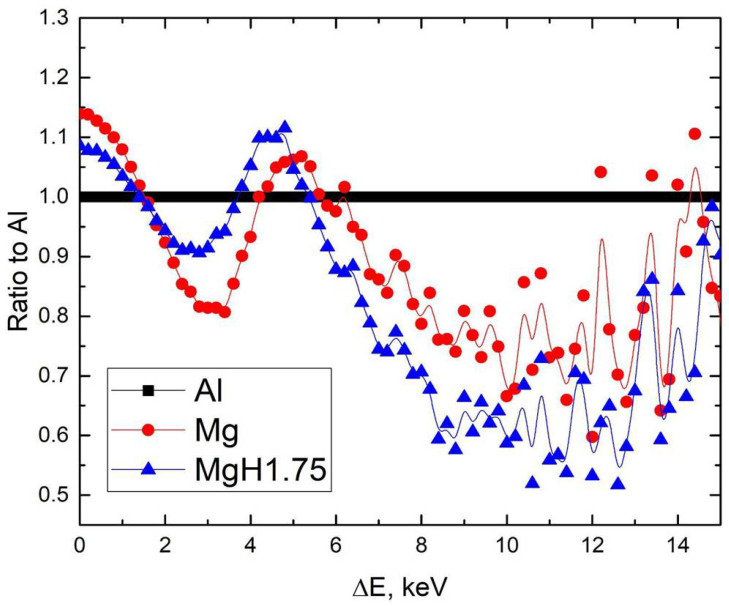
Ratio curves of CDB spectra to Al for milled magnesium and magnesium hydride powders.

Figure 6 shows that magnesium hydride exhibits changes in the low- (<2 keV) and high-energy regions. The decrease in the low-energy region is a direct result of the metal-insulator transition during hydrogenation to the MgH_2_ phase [44]. The remaining difference in high-energy regions can be related to a significant difference in the positron trapping by the powder surface and the nanopores [45].

**Figure 7:**
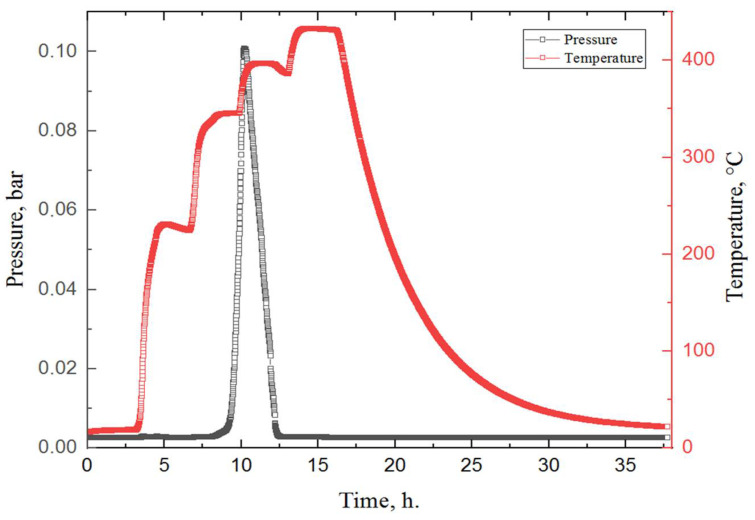
Experimental data obtained during the dehydrogenation process of MgH_2_. Colour code: red—the temperature in the chamber with the sample, according to the heating profile set on the furnace; black—pressure in the chamber.

In the next stage, in situ studies of MgH_2_ during dehydrogenation were carried out. To conduct the dehydrogenation process, milled magnesium hydride was placed in a specially developed Sieverts-type automated apparatus and heated in a vacuum environment. To create a high vacuum, the gas was continuously evacuated from the chamber by a vacuum pump. Figure 7 shows the experimental results of the hydrogen release from magnesium hydride.

According to the results of a hydrogen release study from magnesium hydride, after 11 h in the chamber, one endothermic peak is observed, which corresponds to the release of hydrogen from the material. The beginning of hydrogen desorption from MgH_2_ was observed at a temperature of 345 °C, and the peak temperature was about 380 °C. These results correlate with the data obtained in other works for milled magnesium hydride [46,47,48,49].

During the dehydrogenation process, in situ DBS spectra were acquired to evaluate the state of the defect structure using the isotope ^64^Cu. For comparison, the same measurement was made for magnesium powder without hydrogen.

**Figure 8:**
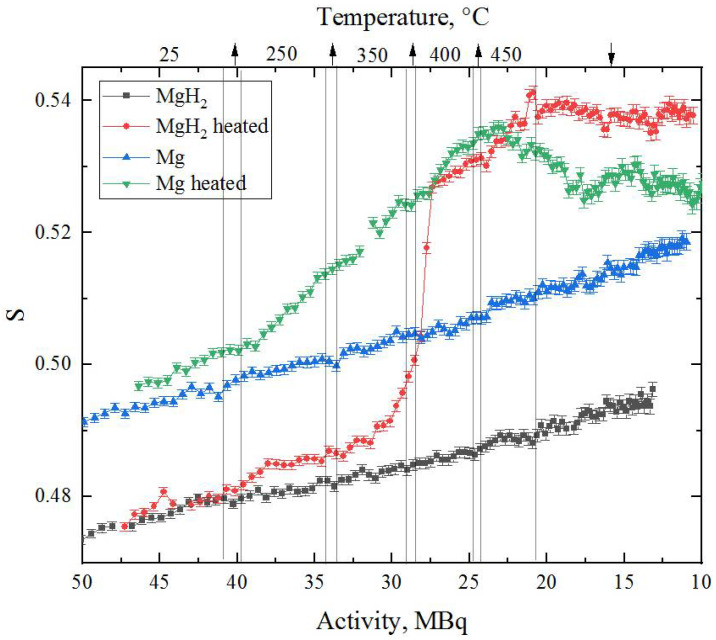
Results of in situ DBS analysis of milled magnesium and magnesium hydride powders during heating and subsequent cooling. The upper scale represents corresponding temperature regions for heated Mg (green) and MgH_2_ (red) at different ^64^Cu source activities (arrows are associated with heating/cooling processes).

The dependence of the line shape parameters of DBS spectra for Mg and MgH_2_ during annealing as well as at room temperature on the activity of ^64^Cu is shown in Figure 8. The dependence of the W parameter is mirrored with respect to the S-parameter, and for this reason is not presented in the paper.

The first thing worth noting is the difference in the S-parameter value for Mg and MgH_2_ at room temperature. The lower value of this parameter in magnesium hydride correlates well with calculated results [43]. It is also known [13] that hydrogen, being localised in the defect, reduces its free volume and, accordingly, affects the characteristics of positron annihilation and the S-parameter.

The constant increase in the S-parameter value with time (at constant temperature) is a consequence of the rapidly changing source activity. This effect was investigated in detail in [21]. In the presented data, it is also clearly seen that the dependence of the S-parameter on activity is smooth and more or less the same (comparing Mg and MgH_2_ without heating), which allows us to compare the results.

It can also be seen from Figure 8 that the green line is slightly above the blue line even at room temperature, even though the material is the same. These deviations are caused by the difference in the pressing level of the studied powders.

The most important result of the in situ studies presented in this work is the behaviour of Mg and MgH_2_ powders during annealing. The rapid growth of the S-parameter during annealing is explained by the formation of thermal vacancies inside the material [21,50,51]. However, if in the case of pure magnesium annealing, the S-parameter growth during heating is accompanied by its subsequent fall to almost the initial value, in magnesium hydride we have a different picture. When the hydride is heated from 350 to 400 °C, there is a sharp increase in the S-parameter value to the line corresponding to pure magnesium. This increase occurs at the same time as the peak of the hydrogen yield from the hydride powder (Figure 7), and is associated with hydride decomposition. At this point, the state of the defect structure comes close to that of the original magnesium at the same temperature. Another distinguishing feature of the temperature annealing of magnesium hydride is that the defect structure after annealing is characterised by a higher free volume than that of the pure Mg powder. Thus, it can be concluded that although the annealing process described in the paper leads to perfect phase coincidence of pure magnesium and hydride, the defect state of these two materials is significantly different. The annealed hydride is characterised by a more developed defect structure, which requires further investigation (including the positron lifetime method). At this stage, it may be assumed that the described effect is due to the formation of more resistant hydrogen-induced defects (such as vacancy–hydrogen complexes).

**Figure 9:**
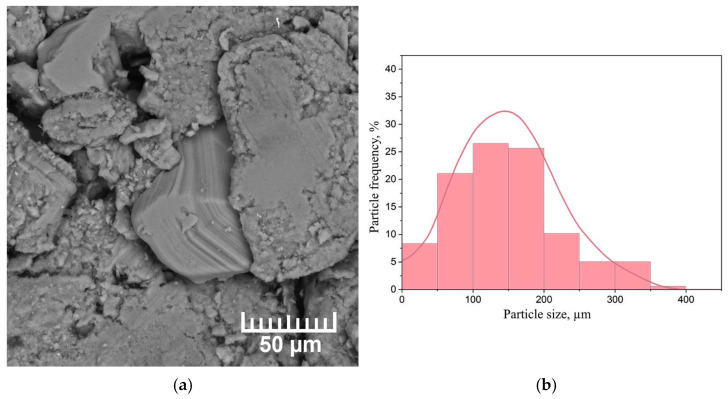
(**a**) SEM micrograph of the powder and (**b**) particle size distribution histogram after MgH_2_ dehydrogenation.

Figure 9 shows a micrograph and particle size distribution histogram of the dehydrogenated MgH_2_ powder.

Comparing magnesium hydride and powder after dehydrogenation, a significant agglomeration of small particles into larger ones is observed. It is worth noting that a small number of magnesium particles began to form crystals. According to the particle size distribution histogram, the average particle size is 150 µm. However, particles with a size less than 20 µm were observed, which did not have time to agglomerate into larger particles.

**Figure 10:**
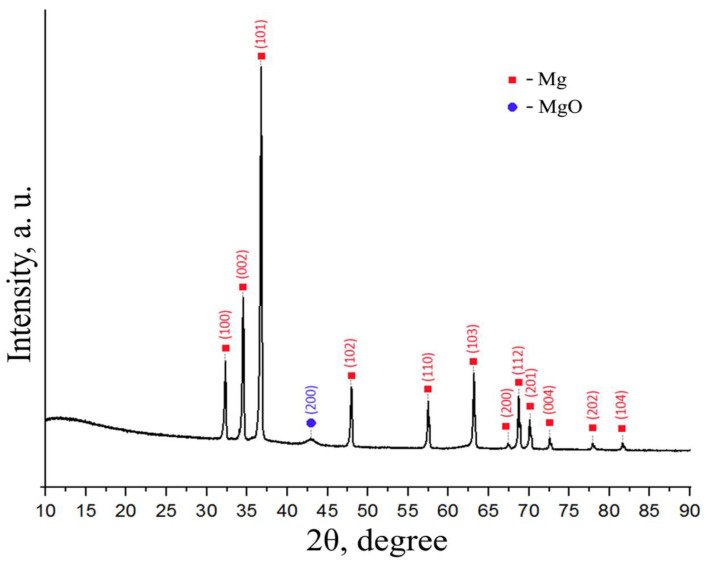
XRD diffraction pattern of magnesium after dehydrogenation.

Figure 10 shows a diffraction pattern for a dehydrated MgH_2_ powder.

Based on the diffraction pattern of the powder obtained after dehydrogenation, the phase composition differs little from that of ground magnesium. Peaks corresponding to Mg with a hexagonal close-packed modification are clearly pronounced. There is also a weak, broad diffraction peak for magnesium oxide. Peaks corresponding to magnesium hydride are not present in the X-ray diffraction pattern.

## 4. Conclusions

A unique digital spectroscopy complex, consisting of a controlled gaseous reactor with a special reaction chamber, a radioisotope positron source, detectors, and spectrometric equipment, has been developed to investigate structural defects in metal–hydrogen systems.

The developed complex is intended for the experimental research of defect structures of solids by methods of positron annihilation spectroscopy, namely PALS and DBS. The developed complex allows measurements of samples located in a vacuum chamber, at elevated temperatures, in a hydrogen atmosphere, and at elevated pressures, including in an in situ mode.

The controlled gas reactor enables monitoring and investigation of the sorption and desorption characteristics in metallic materials in manual and automatic modes up to a temperature of 900 °C and pressure up to 50 bar, including hydrogen and other gases. The above-mentioned characteristics of the developed complex enable high-precision measurements of the electron and defect structure of new advanced hydrogen storage materials under direct operating conditions: namely, in situ sorption and desorption processes.

To validate the capabilities of the developed spectrometric complex, an experimental study of defect structure evolution during vacuum annealing of magnesium powder and magnesium hydride was carried out. The in situ study demonstrated the sensitivity of the shape parameters of the Doppler broadening spectra to the phase state of the material under investigation: a rapid increase in the S-parameter at the moment of hydrogen release, associated with hydride decomposition. It is shown that the defect structure of Mg and MgH_2_ powder after dehydrogenation significantly differs due to the accumulation of irreversible hydrogen-induced defects. Since the development of the defect structure has a direct impact on the sorption characteristics of the material, these changes are key for hydrogen storage materials.

In situ investigations of the defect structure of Mg and MgH_2_ powders correlate well with the results of standard (ex situ) techniques for characterising the initial and final states of the material under investigation.

As a result of this work, a unique digital positron spectrometric complex for the study and control of metal–hydrogen systems, as well as advanced and promising materials, has been created.

## Figures and Tables

**Table 1:** Results of ex situ PALS analysis of milled MPF-4 magnesium after vacuum annealing.

Temperature, °C	τ_A_, ±1 ps	τ_B_, ±2 ps	τ_C_, ±0.02 ns	τ_F_, ±1 ps	k_A_, ±0.01 ns^−1^	k_B_, ±0.01 ns^−1^	k_C_, ±0.01 ns^−1^	I_A_, %	I_B_, %	I_C_, %	τ_avg_, ps
RT	232	392	2.40	226	9.26	0.92	0.94	82.5	7.1	6.2	371
50					8.74	0.66	0.82	84.7	5.4	5.7	359
100					8.19	0.70	0.79	83.6	6.1	5.8	360
150					6.86	0.45	0.66	84.9	4.6	5.5	352
200					6.72	0.55	0.70	83.2	5.6	5.9	360
250					6.08	0.28	0.59	86.1	3.2	5.4	346
300					6.89	0.38	0.73	85.0	3.9	6.1	362
350					3.40	0.53	0.61	73.2	8.3	7.1	386
400					3.04	0.22	0.43	70.0	3.9	5.6	349

## Data Availability

The raw/processed data required to reproduce these findings cannot be shared at this time as the data also forms part of an ongoing study.

## References

[1] Iurii B., Viktor K., Leonid S., Maxim S., Ekaterina S., Jakub Č., Marian V., Ke L., Roman L., Andrey L. (2019). Positron annihilation spectroscopy study of defects in hydrogen loaded Zr-1Nb alloy. J. Alloy. Compd..

[2] Laptev R.S., Lider A.M., Bordulev Y.S., Kudiiarov V.N., Garanin G.V., Wang W., Kuznetsov P.V. (2015). Investigation of Defects in Hydrogen-Saturated Titanium by Means of Positron Annihilation Techniques. Defect and Diffusion Forum.

[3] Laptev R., Lider A., Bordulev Y., Kudiiarov V., Garanin G. (2015). Hydrogenation-induced microstructure changes in titanium. J. Alloy. Compd..

[4] Laptev R.S., Kudiiarov V.N., Bordulev Y.S., Mikhaylov A.A., Lider A.M. (2017). Gas-phase hydrogenation influence on defect behavior in titanium-based hydrogen-storage material. Prog. Nat. Sci. Mater. Int..

[5] Cherdantsev Y.P., Chernov I.P., Tyurin Y.I. (2004). Methods for studying metal-hydrogen systems. Energoatomizdat.

[6] Gel’d P.V., Ryabov R.A. (2014). Hydrogen in metals and alloys. Metallurgy.

[7] Kolachev B.A. (1999). Hydrogen in metals and alloys. Met. Sci. Heat Treat..

[8] Alefeld G., Völkl J. (1978). Hydrogen in Metals I-Basic Properties.

[9] Geld P.V., Ryabov R.A., Kodes E.S. (1979). Hydrogen and imperfections of metal structure. Metallurgy.

[10] Popov E., Troev T., Petrov L., Berovski K., Peneva S., Kolev B. (2015). Model calculations of positron interaction in materials for ITER. Bulg. Chem. Commun..

[11] Gainotti A., Ghezzi C., Manfredi M., Zecchina L. (1968). Positron lifetimes in metal hydrides. Il Nuovo Cimento B Ser. 10.

[12] Budziak A., Dryzek J., Krawczyka J., Zielińskia P.M. (2010). Calorimetric and Positron Lifetime Measurements οf Hydrogenated Carbon Nanocones. Acta Phys. Pol. A.

[13] Laptev R.S., Bordulev Y.S., Kudiiarov V.N., Lider A.M., Garanin G.V. (2014). Positron Annihilation Spectroscopy of Defects in Commercially Pure Titanium Saturated with Hydrogen. Adv. Mater. Res..

[14] Hautojarvi P., Huomo H., Puska M., Vehanen A. (1985). Vacancy recovery and vacancy-hydrogen interaction in niobium and tantalum studied by positrons. Phys. Rev. B.

[15] Kulkova S. (1996). Electron and positron characteristics of group IV metal dihydrides. Int. J. Hydrog. Energy.

[16] Aref’ev K., Oleg B., Olga I., Surkov A.S., Chernov I.P. (2003). Annihilation of positrons in hydrogen-saturated titanium. Phys. Solid State.

[17] Sakaki K., Kawase T., Hirato M., Mizuno M., Araki H., Shirai Y., Nagumo M. (2006). The effect of hydrogen on vacancy generation in iron by plastic deformation. Scr. Mater..

[18] Chernov I.P. (2002). Accumulation and elimination of hydrogen defects under radiation and heat treatment of titanium. Fiz. Khimiya Obrab. Mater..

[19] Takai K., Shoda H., Suzuki H., Nagumo M. (2008). Lattice defects dominating hydrogen-related failure of metals. Acta Mater..

[20] Middleburgh S.C., Voskoboinikov R.E., Guenette M.C., Riley D.P. (2014). Hydrogen induced vacancy formation in tungsten. J. Nucl. Mater..

[21] Bordulev I., Laptev R., Kabanov D., Ushakov I., Kudiiarov V., Lider A. (2021). Source for In Situ Positron Annihilation Spectroscopy of Thermal—And Hydrogen-Induced Defects Based on the Cu-64 Isotope. Materials.

[22] Voyt A.P., Elets D.I., Denisov E.A., Gabis I.E. (2019). Hydrogen release from magnesium hydride subjected to uniaxial pressing. Mater. Sci..

[23] Chen X., Zou W., Lin Q., Li R., Xia G., Yu X. (2019). The effect of oxygen coverages on hydrogenation of Mg (0001) surface. Appl. Surf. Sci..

[24] Sun Y., Shen C., Lai Q., Liu W., Wang D., Aguey-Zinsou K. (2018). Tailoring magnesium based materials for hydrogen storage through synthesis: Current state of the art. Energy Storage Mater..

[25] Banrejee S., Kumar A., Ruz P., Sudarsan V. (2021). Improvement of hydrogen storage characteristics of catalyst free magnesium nanoparticles prepared by wet milling. Int. J. Energy Res..

[26] Luna C.R., Macchi C.E., Juan A., Somoza A. (2010). Electronic and bonding properties of MgH_2_–Nb containing vacancies. Int. J. Hydrog. Energy.

[27] Robles J.M.C., Ogando E., Plazaola F. (2007). Positron lifetime calculation for the elements of the periodic table. J. Phys. Condens. Matter.

[28] Dryzek J., Dryzek E., Suzuki T., Yu R. (2005). Subsurface zone in pure magnesium studied by positron lifetime spectroscopy. Tribol. Lett..

[29] Hautojärvi P. (1982). Trapping of positrons at vacancies in magnesium. Appl. Phys. A.

[30] Checchetto R., Bazzanella N., Kale A., Miotello A., Mariazzi S., Brusa R., Mengucci P., Macchi C., Somoza A., Egger W. (2011). Enhanced kinetics of hydride-metal phase transition in magnesium by vacancy clustering. Phys. Rev. B.

[31] Čížek J., Procházka I., Smola B., Stulíková I., Kužel R., Matěj Z., Cherkaska V., Islamgaliev R.K., Kulyasova O. (2005). Defects in ultra-fine grained Mg and Mg-based alloys prepared by high pressure torsion studied by positron annihilation. Acta Phys. Pol. A.

[32] Skowron K., Wrobel M., Mosiałek M., Le Joncour L., Dryzek E. (2020). Gradient Microstructure Induced by Surface Mechanical Attrition Treatment (SMAT) in Magnesium Studied Using Positron Annihilation Spectroscopy and Complementary Methods. Materials.

[33] Staab T.E.M., Krause-Rehberg R., Vetter B., Kieback B. (1999). The influence of microstructure on the sintering process in crystalline metal powders investigated by positron lifetime spectroscopy: I. Electrolytic and spherical copper powders. J. Phys. Condens. Matter.

[34] Kudiyarov V.N., Elman R.R., Kurdyumov N. (2021). The effect of high-energy ball milling conditions on microstructure and hydrogen desorption properties of magnesium hydride and single-walled carbon nanotubes with iron nanoparticles. Metals.

[35] Baran A., Polański M. (2020). Magnesium-based materials for hydrogen storage—A scope review. Materials.

[36] Juahir N., Mustafa N.S., Sininb A.M., Ismail M. (2015). Improved hydrogen storage properties of MgH_2_ by addition of CO_2_ NiO nanoparticles. RSC Adv..

[37] Mustafa N.S., Law M.C., Ismail M. (2016). Study the effect of NiF_2_ additive on the hydrogen sorption properties of 4MgH_2_+ Li_3_AlH_6_ destabilized system. Mater. Today Proc..

[38] Vigeholm B., Kjøller J., Larsen B. (1980). Magnesium for hydrogen storage. J. Less Common Met..

[39] Bobet J.L., Chevalier B., Song M.Y., Darriet B., Etourneau J. (2002). Hydrogen sorption of Mg-based mixtures elaborated by reactive mechanical grinding. J. Alloy. Compd..

[40] Mirabile Gattia D., Jangir M., Jain I.P. (2020). Behavior of Compacted Magnesium-Based Powders for Energy-Storage Applications. Inorganics.

[41] Vajeeston P., Ravindran P., Kjekshus A., Furuseth S., Hauback B., Fjellvaag H., Hanfland M. (2006). Structural stability and pressure-induced phase transitions in MgH_2_. Phys. Rev. B.

[42] Chawla K., Yadav D., Deepak K., Bajpai A., Kumar S., Lal C. (2021). Hydrogenation properties and kinetic study of MgH_2_-x wt% AC nanocomposites prepared by ball milling. Environ. Sci. Pollut. Res..

[43] Anastasopol A., Eijt S.W.H., Schut H., Mulder F.M., Plazaola F., Dam B. (2012). Thermal stability of MgyTi1-y thin films investigated by positron annihilation spectroscopy. Phys. Procedia.

[44] Eijt S.W.H., Leegwater H., Schut H., Anastasopol A., Egger W., Ravelli L., Hugenschmidt C., Dam B. (2011). Layer-resolved study of the Mg to MgH_2_ transformation in Mg–Ti films with short-range chemical order. J. Alloy. Compd..

[45] Eijt S.W.H., Kind R., Singh S., Schut H., Legerstee W.J., Hendrikx R.W.A., Svetchnikov V.L., Westerwaal R.J., Dam B. (2009). Positron depth profiling of the structural and electronic structure transformations of hydrogenated Mg-based thin films. J. Appl. Phys..

[46] Peng C., Li Y., Zhang Q. (2021). Enhanced hydrogen desorption properties of MgH_2_ by highly dispersed Ni: The role of in-situ hydrogenolysis of nickelocene in ball milling process. J. Alloy. Compd..

[47] Wu Z., Fang J., Liu N., Wu J., Kong L. (2021). The Improvement in Hydrogen Storage Performance of MgH_2_ Enabled by Multilayer Ti_3_C_2_. Micromachines.

[48] Sazelee N.A., Idris N.H., Md Din M., Yahya M.D., Ali N., Ismail M. (2020). LaFeO_3_ synthesised by solid-state method for enhanced sorption properties of MgH_2_. Results Phys..

[49] Jangir M., Meena P., Jain I.P. (2018). Improved hydrogen storage properties of MgH_2_ catalyzed with TiO_2_. AIP Conference Proceedings.

[50] Druzhkov A.P., Nikolaev A.L. (2011). Effects of solute atoms on evolution of vacancy defects in electron-irradiated Fe–Cr-based alloys. J. Nucl. Mater..

[51] Enzinger R., Neubauer C., Kotzurek J., Sprengel W., Würschum R. (2018). Kinetics of vacancy annealing upon time-linear heating applied to dilatometry. J. Mater. Sci..

